# Prognostic significance of c-Met in breast cancer: a meta-analysis of 6010 cases

**DOI:** 10.1186/s13000-015-0296-y

**Published:** 2015-06-06

**Authors:** Shunchao Yan, Xin Jiao, Huawei Zou, Kai Li

**Affiliations:** Department of Oncology, Shengjing Hospital of China Medical University, Shenyang, 110022 China; Department of Respiratory Medicine, Shenyang Chest Hospital, Shenyang, 110044 China

**Keywords:** c-Met, Breast cancer, Meta-analysis, Prognosis

## Abstract

**Background:**

The prognostic value of c-Met in breast cancer remains controversial. A meta-analysis of the impact of c-Met in breast cancer was performed by searching published data.

**Methods:**

Published studies analyzing overall survival (OS) or relapse free survival (RFS) according to c-Met expression were searched. The principal outcome measures were hazard ratios (HRs) for RFS or OS according to c-Met expression. Combined HRs were calculated using fixed- or random- effects models according to the heterogeneity.

**Results:**

Twenty-one studies involving 6,010 patients met our selection criteria. The impact of c-Met on RFS and OS was investigated in 12 and 17 studies, respectively. The meta-analysis results showed that c-Met overexpression significantly predicted poor RFS and OS in unselected breast cancer. Subgroup analysis indicated that c-Met overexpression was correlated with poor RFS and OS in Western patients, but was not associated with RFS or OS in Asian patients. C-Met was associated with poor OS in lymph node negative breast cancer and with poor RFS in hormone-receptor positive and triple negative breast cancer, but was not associated with prognosis in human epidermal growth factor receptor (HER)-2 positive breast cancer.

**Conclusions:**

C-Met overexpression is an adverse prognostic marker in breast cancer, except among Asian and HER-2 positive patients.

**Virtual slides:**

The virtual slide(s) for this article can be found here: http://www.diagnosticpathology.diagnomx.eu/vs/1869780799156041

## Background

Breast cancer is the most common cancer among women worldwide [1]. The clinical application of targeted therapies, such as tamoxifen and trastuzumab, has decreased the mortality of breast cancer in recent years. However, epidemiological studies show that more than 400,000 patients worldwide die from breast cancer each year [2]. Breast cancer is a heterogeneous disease that has been classified into five molecular subtypes: luminal A, luminal B, human epidermal growth factor receptor-2 (HER-2) overexpressing, basal-like, and normal-like [3]. Current therapeutic regimens for breast cancer are designed according to clinical pathological factors and molecular typing. However, patients with the same clinical stage and molecular type often display markedly different treatment responses and overall outcomes, which lead to treatment failure [4–7]. Therefore, the identification of new prognostic factors and potential therapeutic targets is necessary to improve individual treatment strategies.

The tyrosine kinase c-Met, a key regulator of invasive growth, is overexpressed in certain aggressive cancer cells [8]. c-Met, also called MET and hepatocyte growth factor receptor (HGFR), is a plasma membrane protein that transduces signals from the extracellular matrix to the cytoplasm and is activated by binding to HGF [9]. c-Met is involved in uncontrolled survival, growth, angiogenesis and metastasis of cancer cells [10]. Crizotinib, a dual tyrosine kinase inhibitor of ALK and c-Met kinases, has shown promising results in the treatment of lung adenocarcinoma [11]. Tivantinib, a c-Met inhibitor is being tested in patients with MET-high hepatocellular carcinoma in an ongoing Phase III clinical trial [12]. c-Met was shown to be involved in the development of herceptin and endocrine therapy resistance in breast cancer [13, 14]. However, no evidence-based clinical data are available for c-Met inhibitors in breast cancer treatment. Despite the fact that the prognostic role of c-Met in breast cancer has been discussed since the 1990s [15, 16], there is no consensus on its impact. Some studies suggest that c-Met is a stronger prognostic indicator of poor prognosis than traditional markers such as Her2/neu and epidermal growth factor receptor (EGFR) [17–19], whereas others show no statistically significant relation between c-Met and prognosis in breast cancer [20, 21]. In recent years, c-Met was reported to be associated with favorable prognosis in breast cancer patients [22, 23]. Therefore, systematic studies are necessary to obtain high level evidence-based results of the prognostic value of c-Met for the identification of patients who would benefit from c-Met targeted therapy and to guide future clinical trials.

In the present study, we enrolled and combined all eligible published studies analyzing the relationship between c-Met expression and relapse free survival (RFS) or overall survival (OS) in breast cancer to clarify the relationship between c-Met expression and prognosis in breast cancer. c-Met plays a critical role in early-stage invasion of cancer cells [24], and crosstalk of c-Met signaling pathways with estrogen receptor (ER) and HER-2 signaling pathways has been reported [13, 25]. To validate the prognostic role of c-Met in different subtypes breast cancer, we performed a subgroup analysis in lymph node negative and different molecular subtypes of breast cancer.

## Methods

### Search strategy

We searched the electronic databases PubMed, Embase, and the Chinese Biomedical Literature database (CBM) (last search updated in January 1, 2015) by using the keywords “breast cancer”, “hepatocyte growth factor receptor”, “HGFR”, “c-Met”, and “prognosis”. The titles and abstracts of the studies were firstly scanned to exclude all irrelevant papers. Then, the final inclusion of studies was determined by reading the full text of the remaining articles. The citation lists of all retrieved articles were scanned to identify other potentially relevant reports.

### Selection criteria

The search results were screened according to specific inclusion and exclusion criteria as follows. Inclusion criteria: (1) research limited to human primary breast cancer; (2) the study was published in English or Chinese; (3) inclusion of female patients; (4) evaluation of survival information, such as RFS, OS, according to c-Met expression; (5) the study provided the hazard ratios (HRs) and 95 % confidence intervals (CIs), or data that could be used to calculate the HRs and 95 % CIs, or Kaplan–Meier survival curves that provided sufficient data to extract HRs and 95 % CIs; (6) peer-reviewed and published original articles. Exclusion criteria: (1) no data on survival, or inability to calculate the hazard ratios of RFS and OS based on the data provided; (2) letters to editor, reviews and articles published in a book. If patients were enrolled from the same institutions during the same period, the most recently published data were included in the study.

### Data extraction

Two reviewers (Yan SC and Jiao X) performed the search and assessed the studies independently, and the inclusion of a study was decided by consensus. The following items were recorded from each study: the first author’s name, year of publication, language, cohort size, assessment methods of c-Met expression, type of patients, hazard ratio (HR) of OS and/or RFS. The studies were assessed for quality using REMARK (reporting recommendations for tumor MARKer prognostic studies) [26], and the definitions of the 18 items for reporting study quality provided by Chen et al. [27].

### Statistical analysis

HRs with 95 % CIs were combined to determine the effective value. If data on HRs and 95 % CIs were not provided directly, the published data and Kaplan-Meier survival curves were used to calculate the HR according to the methods described by Parmaret et al. [28] and Tierney et al. [29]. By convention, an observed HR >1 implied a worse survival for the group with c-Met overexpression. The χ^2^-square test was used to assess heterogeneity. A *P-*value < 0.05 was considered significant. If the test of heterogeneity was significant, a combined HR was calculated using the random-effects model; otherwise, the fixed-effects model was used. Engauge Digitizer version 2.11 (free software downloaded from http://sourceforge.net) was used to extract data from Kaplan–Meier curves. Data combining was performed using RevMan version 5.2 (free software downloaded from http://www.cochrane.org). Begg’s tests were used to assess publication bias. Probable significant publication bias was considered at *P* < 0.05. In cases of publication bias, the combined estimate was recalculated after imputation from the asymmetry of the funnel plot of the number of “missing” studies, a method known as “trim and fill”. Begg tests and “trim and fill” were performed using StataSE12.0 (Stata Corp LP, College Station, Texas, USA).

## Results

### Description of studies

**Fig. 1:**
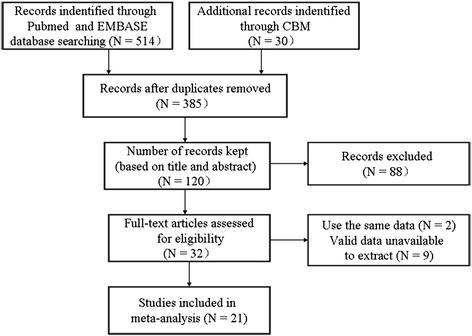
Brief flow chart. N = number of studies; CBM = Chinese Biomedical Literature database

As shown in Fig 1, 544 articles were identified, of which 512 were excluded after screening titles and abstracts because they were irrelevant to this study. Three studies were performed in the same institution during the same period; therefore, the most recent study was included and the remaining two were excluded. Nine articles did not provide HRs and the survival data was not sufficient to calculate HRs (validated data unavailable for extraction). Finally, there were 21 eligible studies published between 1991 and 2014 that satisfied the criteria for our meta-analysis [16–23, 30–42]. Five methods were used for the assessment of c-Met expression in breast cancer specimens as follows: immunohistochemistry (IHC), real-time quantitative PCR (RT-PCR), reverse phase protein lysate microarray (RPPA), fluorescence in situ hybridization (FISH), and molecular inversion probes (MIP). All of the 21 eligible studies were retrospective. Table 1 and Table 2 summarize the characteristics of these studies. The number of patients ranged from 33 to 1002, and the total number of patients analyzed was 6010. Most of the patients included had stage I–IIIa disease and had undergone radical surgery, except one study that included patients with metastatic breast cancer (132 patients) [36].

**Table 1:** Characteristics of the studies included in the meta-analysis

First author	Year	Language	Patients source	Patients Number	Technique	Type of patients	HR estimation	HR(95%CI) of OS	HR(95%CI) of RFS
Ghoussoub	1998	English	USA	88	IHC	BC	Given by author	3.47 (1.22-9.90)	NA
Camp	1999	English	USA	113	IHC	LNN BC	Given by author	5.05 (1.20-21.30)	NA
Nakopoulou	2000	English	Greece	43	IHC	BC	Survival curve	0.14 (0.00-6.41)	NA
Ocal	2003	English	USA	324	IHC	LNN BC	Given by author	2.04 (1.26-3.30)	NA
Kang	2003	English	USA	330	IHC	LNN BC	Given by author	1.86 (1.19-2.91)	NA
Lengyel	2005	English	USA	40	IHC	LNN BC	Given by author	NA	3.00 (1.08-8.30)
Chen	2007	English	Taiwan	104	IHC	Early stage (T1-2N0M0) BC	Given by author	NA	3.33 (1.67-6.65)
Vendrell	2008	English	France	33	RTQ-PCR	ER positive BC	Given by author	1.08 (0.40-2.88)	1.38 (0.60-3.21)
Ponzo	2009	English	Canada	668	IHC	LNN BC	Given by author	NA	1.35 (0.87-2.10)
Liu	2011	Chinese	China	106	IHC	BC	Survival curve	2.41 (0.33-17.71)	NA
Gisterek	2011	English	Poland	302	IHC	BC	Survival curve	0.45 (0.22-0.93)	NA
Li	2012	Chinese	China	100	IHC	BC	Survival curve	1.6 (0.15-17.49)	1.59 (0.54-4.74)
Raghav	2012	English	USA	257	RPPA	BC	Given by author	2.81 (1.19-6.64)	2.06 (1.08-3.94)
Minuti	2012	English	Poland	132	FISH	HER-2 positive MBC	Given by author	1.12 (0.65-1.93)	NA
Gonzalez-Angulo	2013	English	USA	970	MIP	BC	Given by author	NA	1.53 (0.98-2.38)
Zagouri	2013	English	Austria	170	IHC	TNBC	Given by author	3.74 (1.65-8.46)	3.43 (1.65-7.12)
Ho-Yen	2014	English	UK	1002	IHC	BC	Given by author	1.85 (1.07-3.19)	NA
Inanc	2014	English	Turkey	97	IHC	TNBC	Given by author	1.16 (0.55-2.45)	2.05 (0.96-4.37)
Zagouri	2014	English	Austria	78	IHC	ER and HER-2 positive BC	Given by author	1.32 (0.91-1.90)	1.22 (0.91-1.63)
Koh	2014	English	Korea	129	IHC	BC	Given by author	0.37 (0.16-0.86)	0.65 (0.33-1.26)
Kim	2014	English	Korea	924	IHC	BC	Given by author	1.78 (1.26-2.51)	1.39 (1.08-1.78)

*Note*: IHC, immunohistochemistry; RT-PCR, Real-time quantitative PCR; RPRP, Reverse phase protein lysate microarray; FISH, Fluorescence in situ hybridization; MIP, Molecular Inversion Probes; BC, breast cancer; MBC, metatastatic breast cancer; TNBC, triple negative breast cancer; LNN, Lymph Node Negative; OS, over survival; RFS, Relapse-free survival; NA, not available

**Table 2:** Characteristics of the studies according to molecular subtypes

First author	Year	Patients Number	Patient source	Technique	Type of patients	HR estimation	HR(95%CI) of OS	HR(95%CI) of RFS
Vendrell	2008	33	France	PCR	ER positive BC	Given by author	1.08 (0.40-2.88)	1.38 (0.60-3.21)
Ponzo	2009	60	Canada	IHC	Basal-like BC	Given by author	NA	3.02 (0.31-29.30)
		447	Canada	IHC	Nonbasal-like BC	Given by author	NA	1.49 (0.86-2.62)
Raghav	2012	64	USA	RPPA	TNBC	Given by author	NA	2.36 (0.86-6.51)
		140	USA	RPPA	hormone receptor positive BC	Given by author	8.28 (1.10-62.59)	3.44 (1.21-9.81)
Gonzalez-Angulo	2013	173	USA	MIP	TNBC	Given by author	NA	1.33 (0.51-3.43)
		583	USA	MIP	hormone receptor positive BC	Given by author	NA	1.86 (1.07-3.25)
		207	USA	MIP	HER-2 positive BC	Given by author	NA	0.92 (0.29-2.95)
Zagouri	2013	170	Austria	IHC	TNBC	Given by author	3.74(1.65-8.46)	3.43 (1.65-7.12)
Zagouri	2014	78	Austria	IHC	ER and HER-2 positive BC	Given by author	1.32 (0.91-1.90)	1.22 (0.91-1.63)
Inanc	2014	97	Turkey	IHC	TNBC	Given by author	1.15 (0.54-2.44)	2.05 (0.96-4.36)

*Note*: IHC, immunohistochemistry; RPRP, Reverse phase protein lysate microarray; FISH, Fluorescence in situ hybridization; MIP, Molecular Inversion Probes; BC, breast cancer; TNBC, triple negative breast cancer; OS, over survival; RFS, Relapse-free survival; NA, not available.cpc

### Impact of c-Met on the RFS and OS of unselected breast cancer

**Fig. 2:**
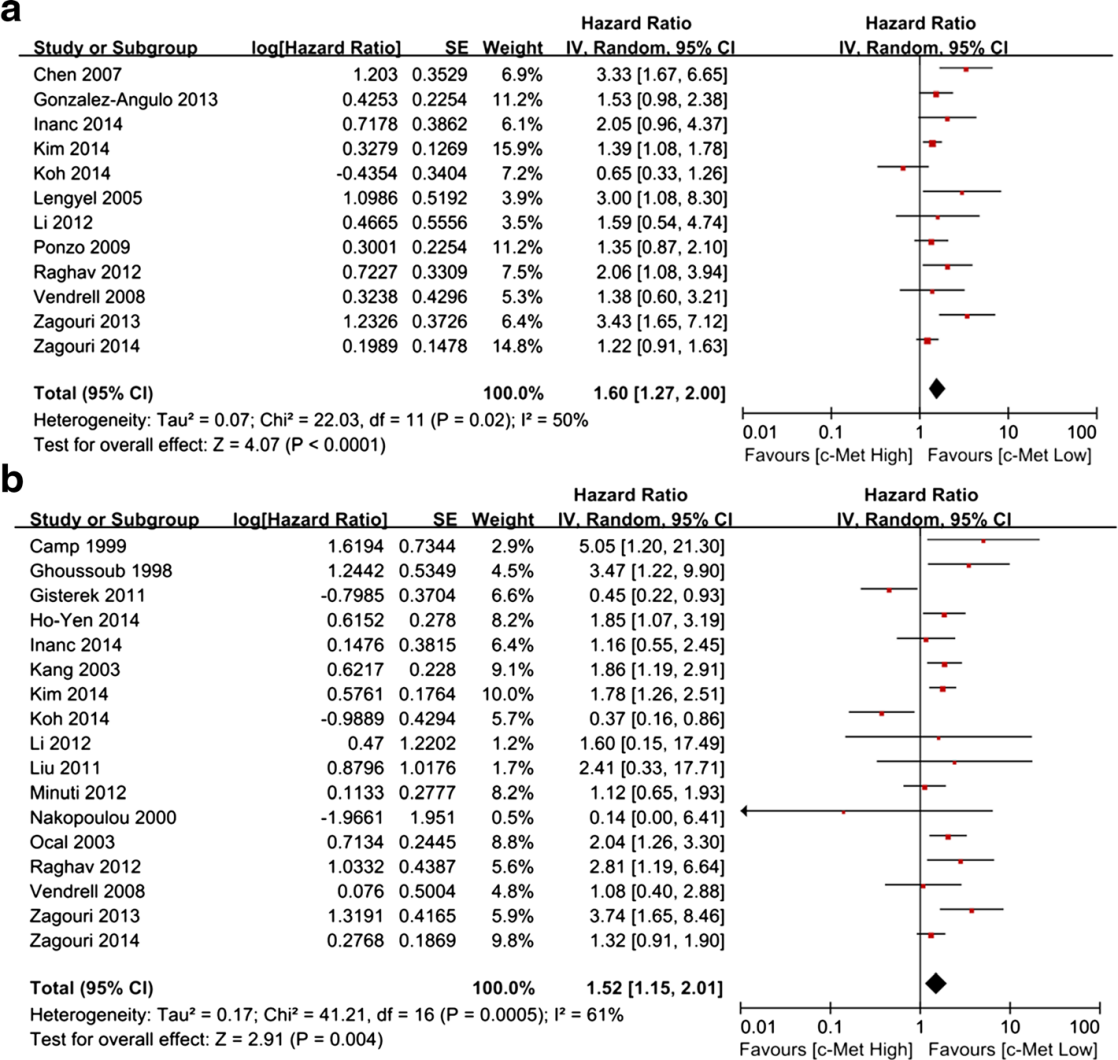
Forest plot of the hazard ratio (HR) for relapse free survival (RFS) (**a**) or overall survival (OS) (**b**) of unselected breast cancer

RFS was analyzed in 12 studies and in a total of 3570 cases. The results showed significant between-study heterogeneity (*P* = 0.02, I^2^ = 50 %), and a random-effects model was used. The combined HR was 1.60 (95 % CI 1.27–2.00; *P* < 0.0001) (Fig. 2a), which indicated that c-Met overexpression was associated with a 1.6-fold increased risk of recurrence. The meta-analysis incorporating the five imputed studies using the trim and fill method still showed a statistically significant poor RFS in c-Met overexpressing patients (HR, 1.28, 95 % CI, 1.01–1.63, *P* = 0.043). Seventeen studies including 4228 cases were evaluated for the effect of c-Met overexpression on OS (Fig. 2b). A random-effects model was used to combine HRs because of the heterogeneity among the studies (*P* = 0.0005; I^2^ = 61 %). The combined HR was 1.52 (95 % CI 1.15–2.01; *P* = 0.004), which indicated that c-Met overexpression was associated with a 1.52-fold increased risk of mortality in breast cancer patients. The trim and fill method omitted one study with a revised estimate of HR and continued to show a statistically significant poor OS in c-Met overexpressing patients (HR, 1.53, 95 % CI, 1.16–2.03, *P* = 0.003).

### Impact of c-Met on the prognosis of Western and Asian patients

**Fig. 3:**
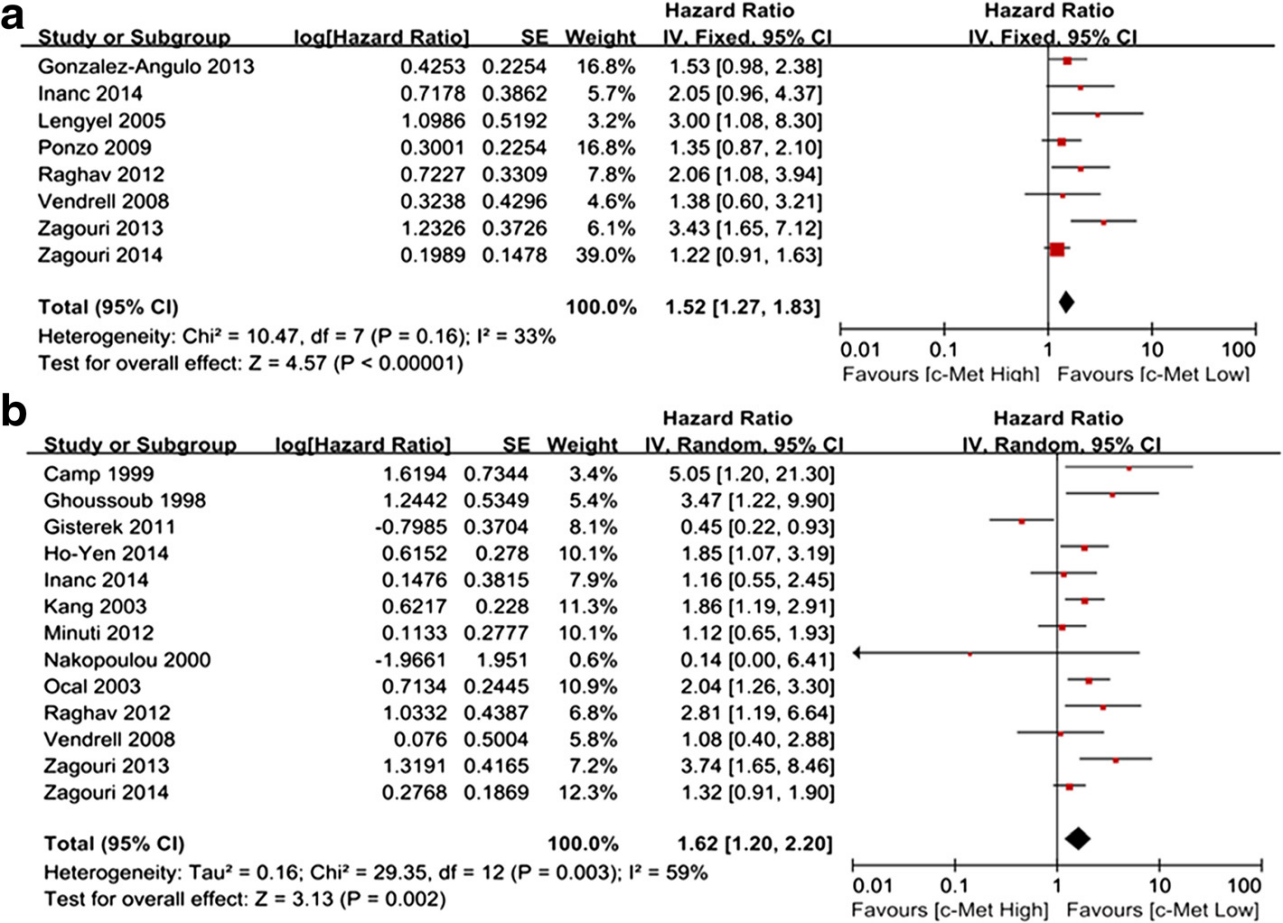
Forest plot of HR for RFS (**a**) and OS (**b**) among Western patients

**Fig. 4:**
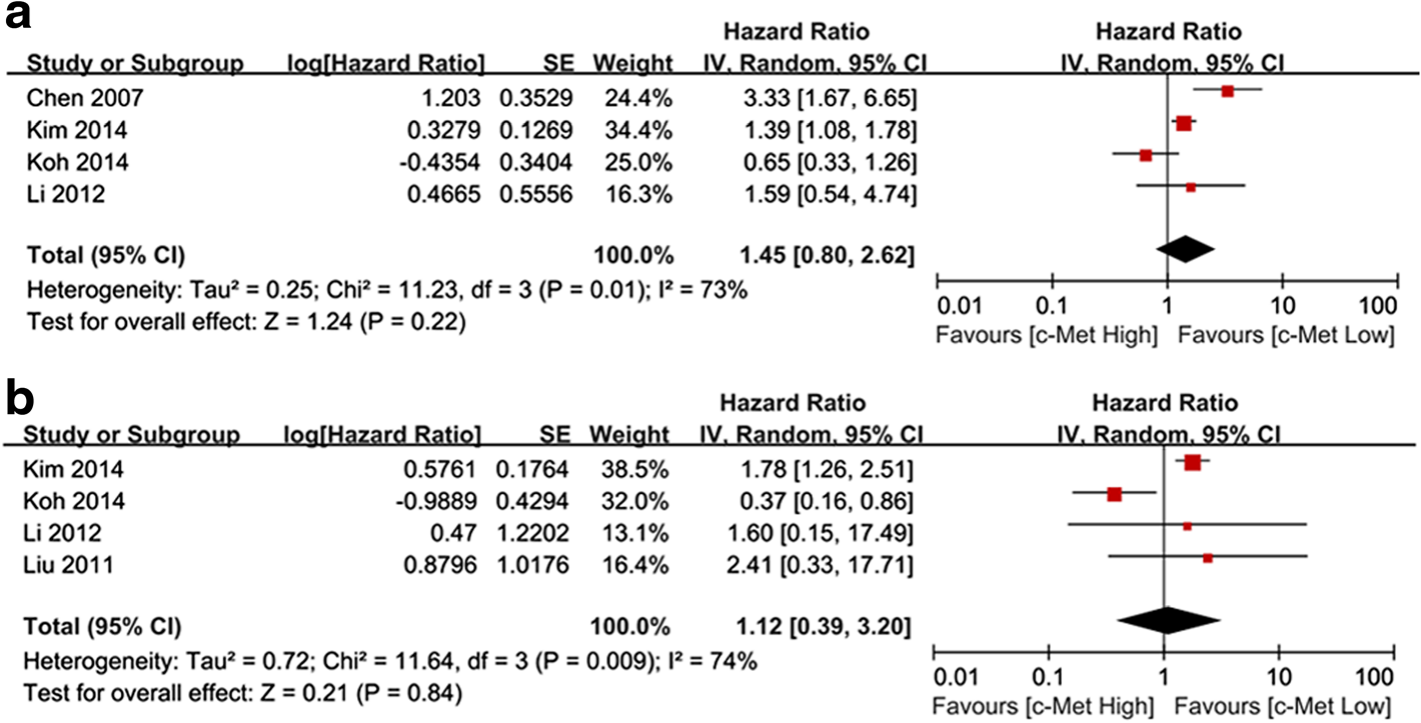
Forest plot of HR for RFS (**a**) and OS (**b**) among Asian patients

In the subgroup analysis according to ethnicity, the impact of c-Met expression on the RFS of Western patients was evaluated in 8 studies including 2313 cases. No significant heterogeneity was observed (*P* = 0.16, I^2^ = 33 %), and the fixed-effects model was used. The results showed that c-Met overexpression was significantly associated with a 1.52-fold increased risk of recurrence (HR = 1.52, 95 % CI 1.27–1.83; *P* < 0.00001) (Fig. 3a). The meta-analysis incorporating the four imputed studies using the trim and fill method still showed a statistically significant poor RFS in c-Met overexpressing patients (HR, 1.32, 95 % CI, 1.12–1.56, *P* = 0.001). The impact of c-Met expression on the OS of Western patients was evaluated in 13 studies including 2969 cases. The random-effects model was used because of the observed heterogeneity (*P* = 0.003, I^2^ = 59 %). The results of the meta-analysis showed a significantly poor OS in the c-Met overexpression group (HR = 1.62, 95 % CI 1.20–2.20, *P* = 0.003) (Fig. 3b). Analysis with the trim and fill method omitted one study and continued to show a statistically significant poor RFS in c-Met overexpressing patients (HR, 1.64, 95 % CI, 1.22–2.22, *P* = 0.001). Four studies including 1257 cases evaluated the impact of c-Met expression on the RFS of Asian patients, and four studies including 1259 cases evaluated the impact of c-Met expression on the OS of Asian patients. The random-effects model was used because of the observed heterogeneity (*P* = 0.01 and 0.009, respectively). Although there was a trend toward increased recurrence (HR 1.45, 95 % CI 0.80–2.62; *P* = 0.22) (Fig. 4a) and mortality (HR 1.12, 95 % CI 0.39–3.20; *P* = 0.84) (Fig. 4b), it was not statistically significant.

### Impact of c-Met on the prognosis of lymph node negative, hormone-receptor positive, HER-2 positive and triple negative breast cancer

**Fig. 5:**
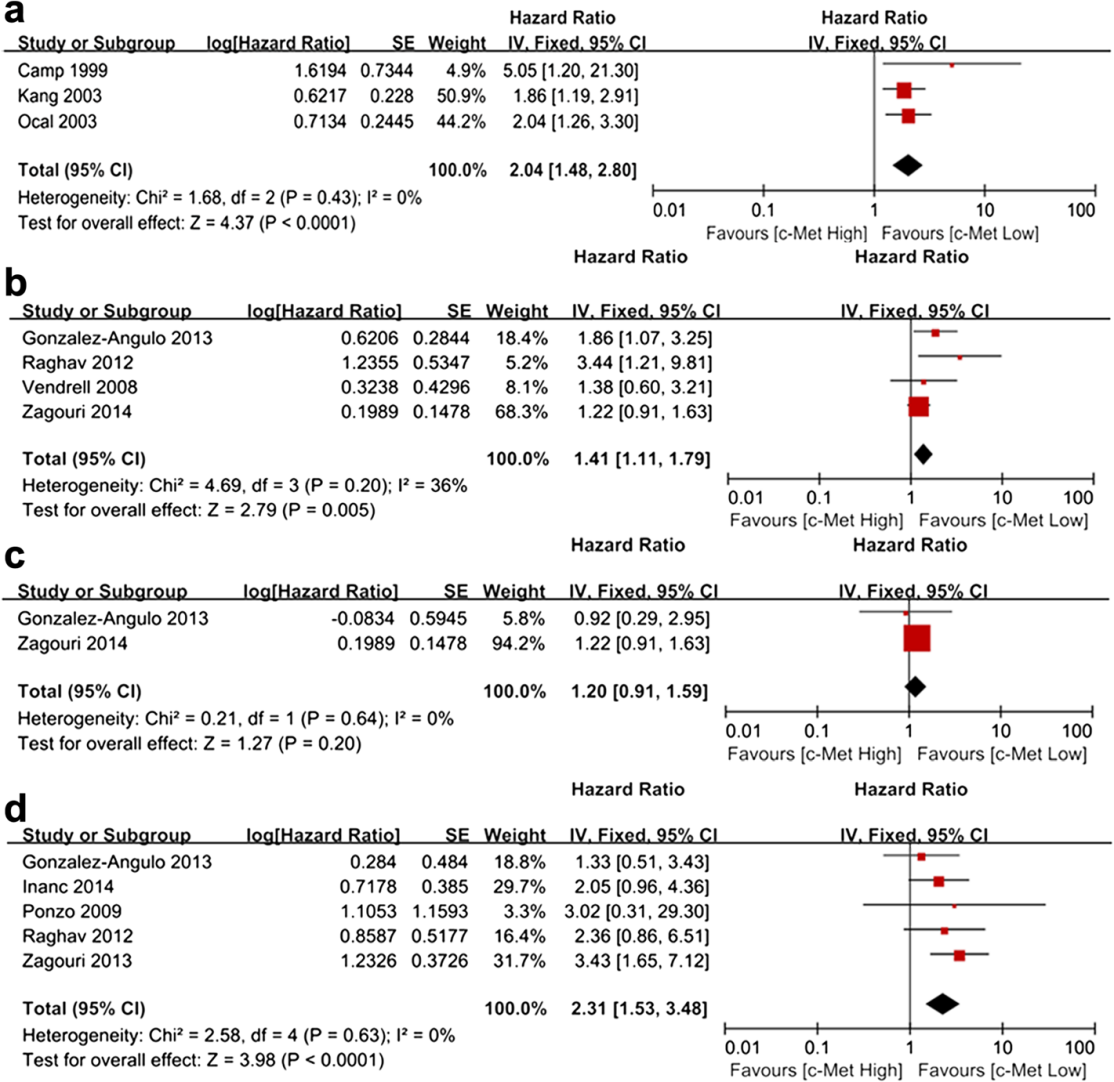
Forest plot of the HR for OS among lymph node negative (**a**), hormone-receptor positive (**b**), HER-2 positive (**c**) and triple negative breast cancer (**d**)

As shown in Table 1, three studies that included lymph node negative patients (767 cases) provided the related OS data. No significant heterogeneity was observed (*P* = 0.43, I^2^ = 0 %), and the fixed-effects model was used. The results showed that c-Met overexpression was associated with a 2.04-fold increased risk of mortality (HR 2.04, 95 % CI 1.48–2.80; *P* < 0.0001) (Fig. 5a). As shown in Table 2, four studies included hormone-receptor positive patients (834 cases) and provided the related RFS data. No significant heterogeneity (*P* = 0.20, I^2^ = 36 %) was observed among these studies. The fixed-effects model was used, and the results of the meta-analysis showed that c-Met overexpression was associated with a 1.41-fold increased risk of recurrence (HR 1.41, 95 % CI 1.11–1.79, *P* = 0.005) (Fig. 5b). Two studies included HER-2 positive patients (285 cases) and provided the related RFS data. The fixed-effects model was used (*P* = 0.64, I^2^ = 0 %). Although there was a trend toward increased recurrence among patients with c-Met overexpression (HR 1.20, 95 % CI 0.91–1.59, *P* = 0.20) (Fig. 5c), it was not statistically significant. The impact of c-Met expression on RFS in patients with triple negative breast cancer (TNBC) was evaluated in five groups including 564 cases. No significant heterogeneity (*P* = 0.63, I^2^ = 0 %) was observed among these studies. The fixed-effects model was used and the result of the meta-analysis showed that c-Met overexpression was significantly associated with a 2.31-fold increased risk of recurrence (HR 2.31, 95 % CI 1.53–3.48, *P* < 0.0001) (Fig. 5d).

### Publication bias

**Fig. 6:**
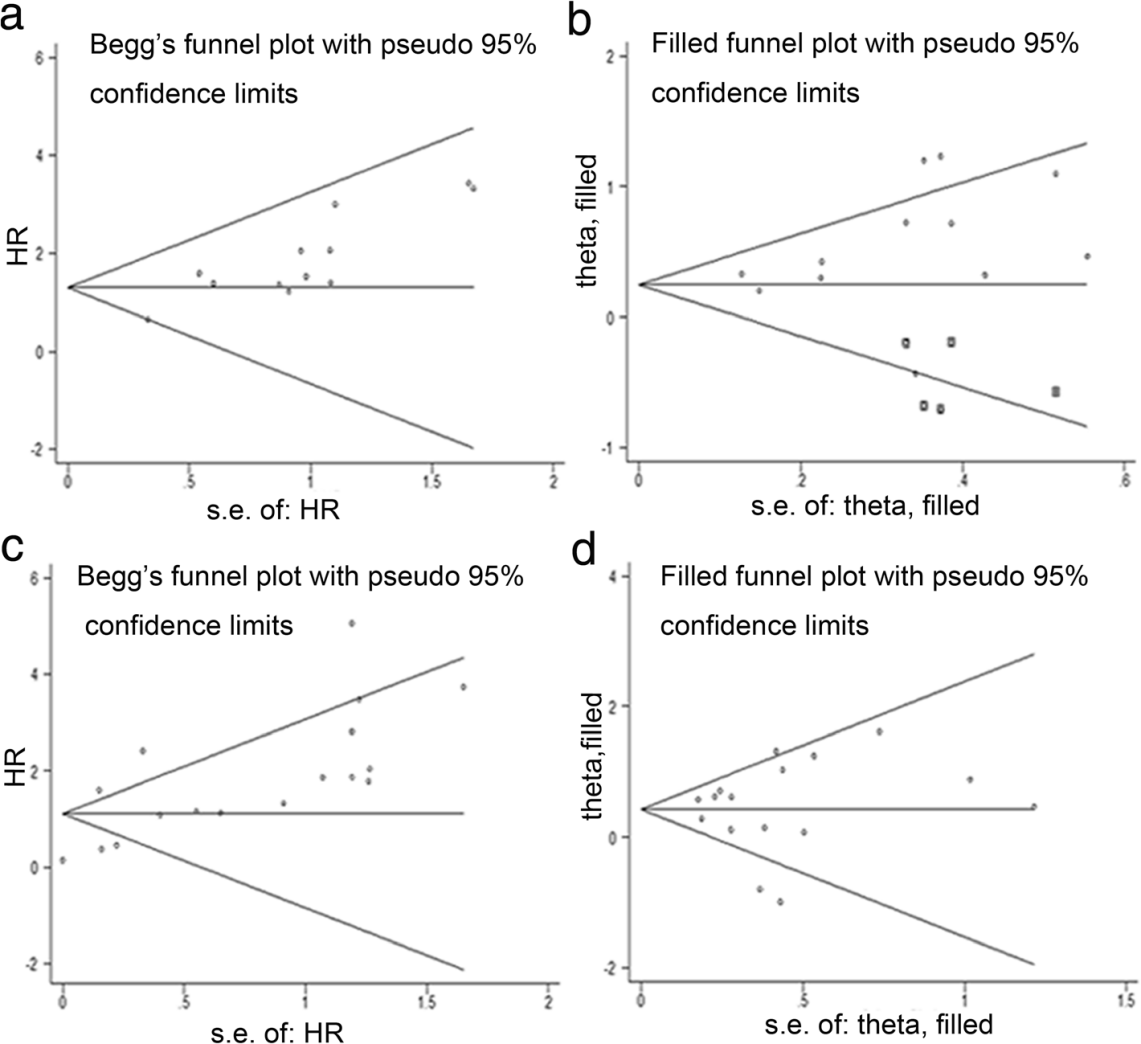
Funnel plot without and with trim and fill for RFS (**a** and **b**) and OS (**c** and **d**) of unselected breast cancer

Twelve studies evaluating RFS in unselected breast cancer patients were examined by Begg’s test. Visual inspection of the funnel plot showed asymmetry (*P* = 0.029) (Fig. 6a), suggesting publication bias. Sensitivity analysis was performed using the trim and fill method, which conservatively imputes hypothetical negative unpublished studies or omits certain studies to mirror the positive studies that cause funnel plot asymmetry. Five hypothetical studies were imputed and the funnel plot symmetry was created (Fig. 6b). The meta-analysis incorporating the imputed studies still showed a statistically significant poor RFS in c-Met overexpressing patients. Seventeen studies evaluating OS in unselected breast cancer patients were analyzed by Begg’s test. Visual inspection of the funnel plot showed asymmetry, although the Begg’s test result was not statistically significant (*P* = 0.105) (Fig. 6c). The trim and fill method omitted one study and created a symmetrical funnel plot (Fig. 6d). The general result was not changed. The Western patient subgroup showed similar results as the unselected breast cancer patients. No publication bias was detected in the other subgroup meta-analyses.

## Discussion

In recent years, the development of target-based therapies has improved the prognosis of cancer patients. However, only a subset of patients benefits from the use of specific drugs, and the development of resistance often results in clinical treatment failure. The identification of novel targets is a challenging task for the medical oncologist, and valuable prognostic markers might become potential therapeutic targets in the future. The trasmembrane tyrosine kinase receptor c-Met plays a vital role in cell survival, growth and metastasis [8]. c-Met is overexpressed in a variety of carcinomas and is associated with resistance to herceptin and gefitinib, and it represents an attractive target for antitumor treatment [13, 43]. c-Met overexpression has been reported in 14–53.6 % of patients with breast cancer [20, 39, 40]. Evidence of the influence of c-Met expression on survival outcomes in breast cancer is inconclusive. In the present study, we analyzed 21 studies published between 1998 and 2014 and comprising a total of 6010 cases. The results of the meta-analysis showed that c-Met overexpression is a statistically significant adverse predictor of RFS and OS in unselected breast cancer. These results provide evidence supporting future trials evaluating the effect of c-Met inhibitors in breast cancer.

Originally, Iressa, a selective EGFR inhibitor, showed promising results among Asian patients, but not in Western populations, suggesting a possible role of ethnic differences between Asian and Western lung cancer patients [44]. The differences in the characteristics of breast cancer between Asian and Western countries have also been discussed for several years [45]. In the present study, we performed a subgroup analysis according to ethnicity. In the Western patient group, there were 8 studies analyzing RFS and 13 studies analyzing OS according to c-Met expression. Our results showed that c-Met is a predictor of poor prognosis (both RFS and OS) in Western patients. In the Asian patient group, four studies analyzing c-Met expression according to OS/RFS were identified. The results showed that there was a trend toward increased recurrence and mortality in c-Met overexpressing patients, although the difference did not reach statistical significance. Further analysis including a larger number of patients and studies is necessary to evaluate the prognostic role of c-Met in Asian breast cancer patients, and to determine whether c-Met status has a different influence on the prognosis of Asian and Western breast cancer patients.

Lymph node status is the best indicator of prognosis in breast cancer. Additional makers are necessary to predict prognosis in patients with lymph node negative breast cancer. C-Met expression is higher and more frequently positive in metastatic lymph nodes than in the primary tumor [19]. In the present analysis, three studies provided data on OS in lymph node negative patients. The meta-analysis results showed that c-Met overexpression was associated with a 2.04-fold increased risk of mortality (combined HR 2.04, 95 % CI 1.48–2.80; *P* < 0.0001) in lymph node negative breast cancer. These results demonstrate that c-Met might act at the early stages of breast cancer, and its expression should be detected on postoperative pathology to predict prognosis and guide the postoperative treatment.

Breast cancer is divided into five molecular subtypes based on the status of ER, PR, HER-2 and Ki67 [3]. In the present study, we performed subgroup analysis according to molecular subtypes. Four studies provided data on RFS in the hormone-receptor positive subgroup. The meta-analysis results showed that c-Met overexpression was associated with a 1.41-fold increased risk of recurrence (combined HR 1.41, 95 % CI 1.11–1.79; *P* = 0.005) in the hormone-receptor positive group. Endocrine therapy is the most important systemic treatment for hormone-receptor positive breast cancer at all stages [46]. C-Met and the Ron receptor tyrosine kinase, a member of the c-Met family of receptors, are associated with resistance to breast cancer endocrine therapy in vitro [14, 47]. Overexpression of HER-2 is associated with resistance to endocrine therapy in breast cancer [48]. Zagouri et al. showed that c-Met was not a prognostic factor in ER- and HER2-positive breast carcinomas [20]. In addition, the prognostic value of c-Met was shown to be independent of HER2/neu [19]. Consequently, c-Met might influence the prognosis of hormone-receptor positive patients by mediating resistance to endocrine therapy, especially in the hormone-receptor positive/HER-2 negative subgroup in a Her-2 independent manner. This subgroup is likely to benefit from combined treatment with c-Met inhibitors and estrogen inhibition therapy in the future. However, additional studies are needed to confirm these results.

Functional crosstalk of c-Met with HER-2 has been reported to enhance cell invasion in Madin-Darby canine kidney (MDCK) epithelial cells in vitro [49]. In breast cancer cells, this crosstalk is involved in the development of Herceptin resistance in vitro [13]. In the present analysis, two studies provided RFS data in HER-2 positive patients. The meta-analysis results showed that c-Met overexpression was associated with poor prognosis, but the findings did not reach statistical significance (combined HR 1.20, 95 % CI 0.91–1.59; *P* = 0.20). Additionally, Minuti et al. found that c-Met is associated with shorter time to progression (TTP) in HER2-positive metastatic breast cancer [36]. Thus, additional studies are necessary to explore the clinical interaction of c-Met and Her-2.

According to currently available data, TNBC is the most aggressive subtype of breast cancer, and no targeted therapy is currently available [39]. TNBC could be further subclassified into basal-like breast cancer (BLBC) and quintuple-negative breast cancer (QNBC), and c-Met is involved in the development of BLBC [33]. Five studies provided RFS data in the TNBC subgroup. The meta-analysis results showed that c-Met overexpression increased recurrence risk by 2.31-fold in TNBC (combined HR 2.31, 95 % CI: 1.53–3.48, *P* < 0.0001), which was the highest risk in this study. The results indicate that c-Met could be a therapeutic target, thereby providing new treatment options for TNBC.

Quality assessment according to REMARK guidelines was performed for all 21 included studies. The studies fulfilled, on average, 14 items (range, 10–18 items) of the guidelines. Sensitivity and sub-group analyses were performed to ensure that the results were reliable and valid. However, our meta-analysis had several limitations. First, the results of sub-analysis were less powerful because the combined HR of some subgroups was calculated on the basis of 2–5 studies with a relative small patient sample size. Second, c-Met was detected by five different methods, although most studies detected c-Met by IHC (excluding the molecular subtype groups). In addition, there were differences in the criteria for c-Met positivity in IHC detection. Third, the funnel plot analysis showed some asymmetry, suggesting the possibility of publication bias in unselected patients and Western patients. The trim and fill sensitivity analysis did not change the general results, suggesting that the results were not influenced by the unpublished negative studies or the small sample size. Additional high-quality data are necessary to draw more reliable conclusions.

## Conclusions

Our comprehensive meta-analysis of all published studies showed that c-Met overexpression is significantly associated with poor survival in breast cancer patients, especially in the TNBC subgroup. In Asian patients and HER-2 positive breast carcinomas, c-Met might not be associated with prognosis.
